# Birth Outcomes and Maternal Residential Proximity to Natural Gas Development in Rural Colorado

**DOI:** 10.1289/ehp.1306722

**Published:** 2014-01-28

**Authors:** Lisa M. McKenzie, Ruixin Guo, Roxana Z. Witter, David A. Savitz, Lee S. Newman, John L. Adgate

**Affiliations:** 1 Department of Environmental and Occupational Health, and; 2 Department of Biostatistics and Informatics, Colorado School of Public Health, Aurora, Colorado, USA; 3 Department of Epidemiology, Brown University, Providence, Rhode Island, USA

## Abstract

Background: Birth defects are a leading cause of neonatal mortality. Natural gas development (NGD) emits several potential teratogens, and U.S. production of natural gas is expanding.

Objectives: We examined associations between maternal residential proximity to NGD and birth outcomes in a retrospective cohort study of 124,842 births between 1996 and 2009 in rural Colorado.

Methods: We calculated inverse distance weighted natural gas well counts within a 10-mile radius of maternal residence to estimate maternal exposure to NGD. Logistic regression, adjusted for maternal and infant covariates, was used to estimate associations with exposure tertiles for congenital heart defects (CHDs), neural tube defects (NTDs), oral clefts, preterm birth, and term low birth weight. The association with term birth weight was investigated using multiple linear regression.

Results: Prevalence of CHDs increased with exposure tertile, with an odds ratio (OR) of 1.3 for the highest tertile (95% CI: 1.2, 1.5); NTD prevalence was associated with the highest tertile of exposure (OR = 2.0; 95% CI: 1.0, 3.9, based on 59 cases), compared with the absence of any gas wells within a 10-mile radius. Exposure was negatively associated with preterm birth and positively associated with fetal growth, although the magnitude of association was small. No association was found between exposure and oral clefts.

Conclusions: In this large cohort, we observed an association between density and proximity of natural gas wells within a 10-mile radius of maternal residence and prevalence of CHDs and possibly NTDs. Greater specificity in exposure estimates is needed to further explore these associations.

Citation: McKenzie LM, Guo R, Witter RZ, Savitz DA, Newman LS, Adgate JL. 2014. Birth outcomes and maternal residential proximity to natural gas development in rural Colorado. Environ Health Perspect 122:412–417; http://dx.doi.org/10.1289/ehp.1306722

## Introduction

Approximately 3.3% of U.S. live-born children have a major birth defect (Centers for Disease Control and Prevention 2013; Parker et al. 2010); these defects account for 20% of infant deaths as well as 2.3% of premature death and disability (McKenna et al. 2005). Oral clefts, neural tube defects (NTDs), and congenital heart defects (CHD) are the most common classes of birth defects (Parker et al. 2010). These defects are thought to originate in the first trimester as a result of polygenic inherited disease or gene–environment interactions (Brent 2004). Suspected nongenetic risk factors for these birth defects include folate deficiency (Wald and Sneddon 1991), maternal smoking (Honein et al. 2006), alcohol abuse and solvent use (Romitti et al. 2007), and exposure to benzene (Lupo et al. 2010b; Wennborg et al. 2005), toluene (Bowen et al. 2009), polycyclic aromatic hydrocarbons (PAHs) (Ren et al. 2011), and petroleum-based solvents, including aromatic hydrocarbons (Chevrier et al. 1996). Associations between air pollution [volatile organic compounds (VOCs), particulate matter (PM), and nitrogen dioxide (NO_2_)] and low birth weight and preterm birth have been reported (Ballester et al. 2010; Brauer et al. 2008; Dadvand et al. 2013; Ghosh et al. 2012; Llop et al. 2010). Many of these air pollutants are emitted during development and production of natural gas (referred to herein as NGD), and concerns have been raised that they may increase risk of adverse birth outcomes and other health effects (Colborn et al. 2011; McKenzie et al. 2012). Increased prevalence of low birth weight and small for gestational age and reduced APGAR scores were reported in infants born to mothers living near NGD in Pennylvania (Hill 2013).

Technological advances in directional drilling and hydraulic fracturing have resulted in a global boom of drilling and production of natural gas reserves [U.S. Energy Information Administration (EIA) 2011a, 2011b; Vidas and Hugman 2008]. NGD is an industrial process resulting in potential worker and community exposure to multiple environmental stressors (Esswein et al. 2013; King 2012; Witter et al. 2013). Diesel-powered heavy equipment is used for worksite development as well as transporting large volumes of water, sand, and chemicals to sites and for waste removal (Witter et al. 2013). It is increasingly common for NGD to encroach on populated areas, potentially exposing more people to air and water emissions as well as to noise and community-level changes that may arise from industrialization [Colorado Oil and Gas Conservation Commission (COGCC) 2009]. Studies in Colorado, Texas, Wyoming, and Oklahoma have demonstrated that NGD results in emission of VOCs, NO_2,_ sulfur dioxide (SO_2_), PM, and PAHs from either the well itself or from associated drilling processes or related infrastructure (i.e., drilling muds, hydraulic fracturing fluids, tanks containing waste water and liquid hydrocarbons, diesel engines, compressor stations, dehydrators, and pipelines) (CDPHE 2007; Frazier 2009; Kemball-Cook et al. 2010; Olaguer 2012; Walther 2011; Zielinska et al. 2011). Some of these pollutants, such as toluene, xylenes, and benzene, are suspected teratogens (Lupo et al. 2010b; Shepard 1995) or mutagens (Agency for Toxic Substances and Disease Registry 2007) and are known to cross the placenta (Bukowski 2001), raising the possibility of fetal exposure to these and other pollutants resulting from NGD. Currently, there are few studies on the effects of air pollution or NGD on birth outcomes.

In this analysis, we explored the association between maternal exposure to NGD and birth outcomes, using a data set with individual-level birth data and geocoded natural gas well locations. We conducted a retrospective cohort study to investigate the association between density and proximity of natural gas wells within a 10-mile radius of maternal residences in rural Colorado and three classes of birth defects, preterm birth, and fetal growth.

## Methods

*Study population*. We used information available in the publically accessible Colorado Oil and Gas Information System (COGIS) to build a geocoded data set with latitude, longitude, and year of development (1996–2009) for all gas wells in rural Colorado (COGIS 2011). Live birth data were obtained from the Colorado Vital Birth Statistics (CDPHE, Denver, CO). Geocoded maternal addresses at time of birth were linked to the well locations. Distance of each maternal residence from all existing (not abandoned) natural gas wells within a 10-mile radius was then computed using spherically adjusted straight line distances. We conducted our analysis on the final de-identified database containing maternal and birth outcome data described below and distance to all wells within the 10-mile radius. The Colorado Multiple Institutional Review Board reviewed and approved our study protocol. Informed consent was not required.

We restricted analysis to births occurring from 1996 through 2009 to focus our analysis on growth of unconventional NGD, characterized by use of hydraulic fracturing and/or directional drilling (King 2012), which expanded rapidly in Colorado beginning around 2000 (COGIS 2011). We also restricted our analysis to rural areas and towns with populations of < 50,000 (excluding the Denver metropolitan area, El Paso County, and the cities of Fort Collins, Boulder, Pueblo, Grand Junction, and Greeley) in 57 counties to reduce potential for exposure to other pollution sources, such as traffic, congestion, and industry. The final study area included locations with and without NGD. We conducted a retrospective study on the resulting cohort of 124,842 live births to explore associations between birth outcomes and exposure to NGD operations. We restricted eligibility to singleton births and excluded the small proportion (< 5%) of nonwhite births because there were too few to analyze separately.

*Birth outcomes*. Identified birth outcomes were *a*) oral cleft, including cleft lip with and without cleft palate as well as cleft palate [*International Classification of Diseases, Ninth Revision, Clinical Modification* (ICD-9-CM) code 749.xx] (National Center for Health Statistics 2011); *b*) NTD, including anencephalus, spina bifida without anecephaly, and encephalocele (ICD-9-CM 740.xx, 741.xx, and 742.0); *c*) CHD, including transposition of great vessels, tetralogy of Fallot, ventricular septal defect, endocardial cushion defect, pulmonary valve atresia and stenosis, tricuspid valve atresia and stenosis, Ebstein’s anomaly, aortic valve stenosis, hypoplastic left heart syndrome, patent ductus arteriosis, coarctation of aorta, and pulmonary artery anomalies (codes 745.xx, 746.xx, 747.xx, excluding 746.9, 747.5); *d*) preterm birth (< 37 weeks completed gestation); *e*) term low birth weight (≥ 37 weeks completed gestation and birth weight < 2,500 g); and *f*) term birth weight as a continuous measure. Births with an oral cleft, NTD, or CHD were excluded from preterm birth and term low birth weight analysis. Preterm births were excluded from term birth weight analysis. Oral cleft, CHD, and NTD cases in the Colorado Responds to Children with Special Needs (CRCSN) birth registry, obtained from hospital records, the Newborn Genetics Screening Program, the Newborn Hearing Screening Program, laboratories, physicians, and genetic, developmental, and other specialty clinics (CRCSN 2011) were matched with Colorado live birth certificates. Cases are reflective of reporting as of 12 July 2012, were not necessarily confirmed by medical record review, and are subject to change as CRCSN ascertains diagnosis up to 3 years of child’s age and/or supplements information by medical record review. We analyzed birth defects in three heterogeneous groups to increase statistical power. Data set information was not sufficient to distinguish between multiple and isolated birth anomalies or to identify chromosomal birth anomalies. In an exploratory analysis, we considered seven clinical diagnostic groupings of CHDs: *a*) conotruncal defects (tetralogy of Fallot and transposition of great vessels); *b*) endocardial cushion and mitrovalve defects (EMD; endocardial cushion defect and hypoplastic left heart syndrome); *c*) pulmonary artery and valve defects (PAV; pulmonary valve atresia and stenosis and pulmonary artery anomalies); *d*) tricuspid valve defects (TVD; tricuspid valve atresia and stenosis and Ebstein’s anomaly); *e*) aortic artery and valve defects (aortic valve stenosis and coarctation of aorta); *f*) ventricular septal defects (VSD); and *g*) patent ductus arteriosis in births > 2,500 g (Gilboa et al. 2005).

*Exposure assessment*. Distribution of the wells within a 10-mile radius of maternal residence shows 50% and 90% of wells to be within 2.3 and 7.7 miles of maternal residence, respectively. We used an inverse distance weighted (IDW) approach, commonly used to estimate individual air pollutant exposures from multiple fixed locations (Brauer et al. 1998; Ghosh et al. 2012), to estimate maternal exposure. Our IDW well count accounts for the number of wells within the 10-mile radius of the maternal residence, as well as distance of each well from the maternal residence, giving greater weight to wells closest to the maternal residence. For example, an IDW well count of 125 wells/mile could be computed from 125 wells each located 1 mile from the maternal residence or 25 wells each located 0.2 miles from the maternal residence. We calculated the IDW well count of all existing natural gas wells in the birth year within a 10-mile radius of each maternal residence as a continuous exposure metric:


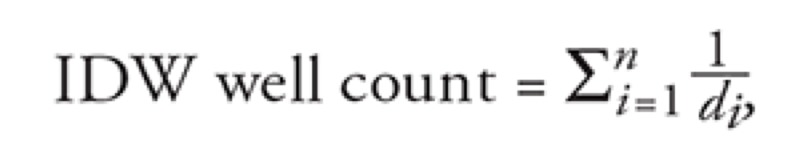
[1]

where IDW well count is the IDW count of existing wells within a 10-mile radius of maternal residence in the birth year; *d_i_* is the distance of the *i*th individual well from maternal residence; and *n* is the number of existing wells within a 10-mile radius of maternal residence in the birth year.

The IDW well count was calculated for each maternal residence with ≥ 1 gas wells within 10 miles. The final distribution then was divided into tertiles (low, medium, and high) for subsequent logistic and linear regression analysis. Each tertile was compared with the referent group (no natural gas wells within 10 miles, IDW well count = 0).

*Statistical analysis*. We used logistic regressions to study associations between each dichotomous outcome and IDW exposure group. We also considered term birth weight as a continuous outcome using multiple linear regression. First, we estimated the crude odds ratio (OR) associated with IDW exposure tertile for each binary outcome, followed by a Cochran–Armitage test to evaluate linear trends in binominal proportions with increasing IDW exposure (none, low, medium, and high). We further investigated associations by adjusting for potential confounders, as well as infant and maternal covariates selected based on both *a priori* knowledge and empirical consideration of their association with exposure and an outcome. Specifically, covariates in our analysis of all outcomes except outcomes with very few events (i.e., NTDs, conotruncal defects, EMDs, and TVDs) included maternal age, education (< 12, 12, 13–15, ≥ 16 years), tobacco use (smoker, nonsmoker), ethnicity (Hispanic, non-Hispanic white), and alcohol use (yes, no), as well as parity at time of pregnancy (0, 1, 2, > 2) and infant sex. Gestational age was also included in the analysis of term birth weight. Elevation of maternal residence also was considered in the analysis because most wells are < 7,000 feet, and elevation has been associated with both preterm birth and low birth weight (Niermeyer et al. 2009). For 272 births where elevation of maternal residence was missing, elevation was imputed using mean elevation for maternal ZIP code. For outcomes with very few events, only elevation was included in the multiple logistic modeling to avoid unstable estimates. The ORs and their 95% CIs were used to approximate relative risks for each outcome associated with IDW count exposure tertile (low, medium, and high) compared with no wells within 10 miles, which is reasonable because of the rarity of the outcomes. We considered the statistical significance of the association, as well as the trend, in evaluating results, at an alpha of 0.05. We evaluated the confounding potential of the 1998 introduction of folic acid fortification on the birth defect outcomes and found only a decrease in NTD prevalence after 1998 (see Supplemental Material, Table S1).

In a sensitivity analyses, we explored reducing exposure to 2- and 5-mile buffers around the maternal residence, as well as restricting the cohort to births occurring between 2000 and 2009 to exclude births before the expansion of NGD. We report estimated associations with 95% CIs. All statistical analyses were conducted using SAS® software version 9.3 (SAS Institute Inc., Cary, NC).

## Results

Births were approximately evenly divided between exposed and unexposed groups (0 wells in a 10-mile radius versus ≥ 1 well in a 10-mile radius) (Table 1). Estimated exposure, represented by IDW well counts, tended to be higher for births to mothers with residence addresses at lower elevations (< 6,000 feet), and among nonsmoking and Hispanic mothers (Table 1).

**Table 1:** Study population characteristics for unexposed and exposed subjects from rural Colorado 1996–2009.

Maternal or infant characteristic	Total	Referent group (0 wells within 10 miles)	Low (first tertile)^*a*^	Medium (second tertile)^*a*^	High (third tertile)^*a*^
Total *n* (%)	124,842	66,626 (53)	19,214 (15)	19,209 (15)	19,793 (16)
Median	27	27	26	27	27
25th percentile	22	22	21	22	23
75th percentile	32	32	30	31	31
Maternal ethnicity (%)^*b*^
Non-Hispanic white	73	74	72	76	69
Sex (%)
Male	51	51	51	51	51
Maternal smoking (%)^*c*^
Smokers	11	11	14	13	8
Maternal alcohol (%)^*c*^
No	99	98	99	99	99
Parity (%)
0	33	33	31	32	32
1	23	23	24	24	25
2	19	19	20	19	20
> 2	25	25	26	25	24
Residential elevation (feet)
Median	5,000–5,999	6,000–6,999	< 5,000	5,000–5,999	< 5,000
25th percentile	< 5,000	5,000–5,999	< 5,000	< 5,000	< 5,000
75th percentile	7,000–7,999	7,000–7,999	5,000–5,999	6,000–6,999	5,000–5,999
Maternal education (%)
< 12 years	21	20	26	19	22
12 years	30	30	33	29	28
13–15 years	23	22	25	25	24
≥ 16 years	26	28	18	26	27
^***a***^First tertile, 1–3.62 wells/mile; second tertile, 3.63–125 wells/mile; third tertile, 126–1,400 wells/mile. ^***b***^Includes both Non-Hispanic and Hispanic white. ^***c***^During pregnancy.					

Both crude and adjusted estimates indicate a monotonic increase in the prevalence of CHDs with increasing exposure to NGD, as represented by IDW well counts (Table 2). Births to mothers in the most exposed tertile (> 125 wells/mile) had a 30% greater prevalence of CHDs (95% CI: 1.2, 1.5) than births to mothers with no wells within a 10-mile radius of their residence.

**Table 2:** Association between inverse distance weighted well count within 10-mile radius of maternal residence and CHDs, NTDs, and oral clefts.

Inverse distance weighted well count^*a*^	0 wells within 10 miles	Low (first tertile)	Medium (second tertile)	High (third tertile)	Cochran–Armitage trend test *p*-value^*b*^
Live births (*n*)	66,626	19,214	19.209	19,793
CHDs
Cases (*n*)	887	281	300	355
Crude OR	1	1.1	1.2	1.3	< 0.0001
Adjusted OR (95% CI)^*c*^		1.1 (0.93, 1.3)	1.2 (1.0, 1.3)	1.3 (1.2, 1.5)
NTDs
Cases (*n*)	27	6	7	19
Crude OR	1	0.77	0.90	2.4	0.01
Adjusted OR (95% CI)^*d*^		0.65 (0.25, 1.7)	0.80 (0.34, 1.9)	2.0 (1.0, 3.9)
Oral clefts
Cases (*n*)	139	31	41	40
Crude OR	1	0.77	1	0.97	0.9
Adjusted OR (95% CI)^*c*^		0.65 (0.43, 0.98)	0.89 (0.61, 1.3)	0.82 (0.55, 1.2)
^***a***^First tertile, 1–3.62 wells/mile; second tertile, 3.63–125 wells/mile; third tertile, 126–1,400 wells/mile. ^***b***^Performed as two-tailed test on unadjusted logistic regression. ^***c***^Adjusted for maternal age, ethnicity, smoking, alcohol use, education, and elevation of residence, as well as infant parity and sex. ^***d***^Adjusted only for residence elevation because of low numbers.					

Prevalence of NTDs was positively associated with only the third exposure tertile, based on crude and estimated adjusted ORs for elevation (Table 2). Births in the highest tertile (> 125 wells/mile) were 2.0 (95% CI: 1.0, 3.9) times more likely to have a NTD than those with no wells within a 10-mile radius, based on 59 available cases. We observed no statistically significant associations between oral clefts and NGD, based on trend analysis across categorical IDW well count exposure (Table 2).

Both crude and adjusted estimates for preterm birth suggest a slight (< 10%) decreased risk of preterm birth with increasing exposure to NGD (Table 3). Crude term low birth weight measures suggested decreased risk of term low birth weight with increasing exposure to NGD. A weak nonlinear trend remained after adjusting for elevation and other covariates. This association is consistent with the multiple linear regression results for continuous term birth weight, in which mean birth weights were 5–24 g greater in the higher IDW well count exposure tertiles than the referent group.

**Table 3:** Association between inverse distance weighted well count within 10-mile radius of maternal residence and preterm birth and term low birth weight.

Inverse distance weighted well count^*a*^	0 wells within 10 miles	Low (first tertile)	Medium (second tertile)	High (third tertile)	Cochran–Armitage trend test *p*-value^*b*^
Preterm birth
Live births (*n*)	65,506	18,884	18,854	19,384
Cases (*n*)	4,849	1,358	1,289	1,274
Crude OR	1	0.97	0.92	0.88	< 0.0001
Adjusted OR (95% CI)^*c*^		0.96 (0.89, 1.0)	0.93 (0.87, 1.0)	0.91 (0.85, 0.98)
Term low birth weight
Full-term live births (*n*)	60,653	17,525	17,565	18,104
Cases (*n*)	2,287	525	471	432
Crude OR	1	0.79	0.70	0.62	< 0.0001
Adjusted OR (95% CI)^*c*^		1.0 (0.9, 1.1)	0.86 (0.77, 0.95)	0.9 (0.8, 1)
Mean difference in birth weight (g)^*d*^	0	5 (–2.2, 13)	24 (17, 31)	22 (15, 29)
^***a***^First tertile, 1–3.62 wells/mile; second tertile, 3.63–125 wells/mile; third tertile, 126–1,400 wells/mile. ^***b***^Performed as two-tailed test on unadjusted logistic regression.^***c***^Adjusted for maternal age, ethnicity, smoking, alcohol use, education, and elevation of residence, as well as infant parity and sex. ^***d***^Adjusted for maternal age, ethnicity, smoking, alcohol use, education, and elevation of residence, as well as infant parity, sex, and gestational age.					

We observed a monotonic increase in the prevalence of NTDs with increasing exposure to NGD in our sensitivity analyses using 2- and 5-mile exposure radii as well as some attenuation in decreased risk for preterm birth and term low birth weight (see Supplemental Material, Tables S2–7). Restricting births to 2000 through 2009, the period of most intense NGD in Colorado, attenuated the positive association between NTDs in the highest tertile and did not alter observed relationships for other birth outcomes (see Supplemental Material, Tables S2–S7).

Exploratory analysis of CHDs by clinical diagnostic groups indicates increased prevalence of PAV defects by 60% (95% CI: 1.1, 2.2), VSDs by 50% (95% CI: 1.1, 2.1), and TVDs by 400% (95% CI: 1.3, 13) in the most exposed tertile compared with those with no wells within a 10-mile radius (Table 4).

**Table 4:** Association between inverse distance weighted well count within 10-mile radius of maternal residence and CHD diagnostic groups.

Inverse distance weighted well count^*a*^	0 wells within 10 miles	Low (first tertile)	Medium (second tertile)	High (third tertile)
Conotruncal defects
Cases (*n*)	40	14	13	15
Adjusted OR (95% CI)^*b*^	1	1.1 (0.57, 2.2)	1.1 (0.55, 2.0)	1.2 (0.6, 2.2)
Ventricular septal defects
Cases (*n*)	210	68	59	84
Adjusted OR (95% CI)^*c*^	1	1.3 (0.96, 1.8)	1.1 (0.81, 1.5)	1.5 (1.1, 2.1)
Endocardial cushion and mitrovalve defects
Cases (*n*)	39	14	12	12
Adjusted OR (95% CI)^*b*^		0.81 (0.42, 1.6)	0.80 (0.41, 1.5)	0.67 (0.33, 1.32)
Pulmonary artery and valve defects
Cases (*n*)	137	52	62	66
Adjusted OR (95% CI)^*c*^	1	1.3 (0.89, 1.8)	1.5 (1.1, 2,1)	1.6 (1.1, 2,2)
Tricuspid valve defects
Cases (*n*)	9	5	8	8
Adjusted OR (95% CI)^*b*^	1	2.6 (0.75, 9.1)	3.9 (1.3, 11)	4.2 (1.3, 13)
Aortic artery and valve defects
Cases (*n*)	75	22	21	24
Adjusted OR (95% CI)^*c*^	1	1.1 (0.68, 1.9)	1.0 (0.62, 1.8)	1.2 (0.73, 2.1)
Patent ductus arteriosis
Cases (*n*)	59	18	17	15
Adjusted OR (95% CI)^*c*^	1	1.0 (0.56, 1.8)	0.96 (0.55, 1.7)	0.83 (0.44, 1.5)
^***a***^First tertile, 1–3.62 wells/mile; second tertile, 3.63–125 wells/mile; third tertile, 126–1,400 wells/mile. ^***b***^Adjusted only for residence elevation of because of low numbers. ^***c***^Adjusted for maternal age, ethnicity, smoking, alcohol use, education, and elevation of residence, as well as infant parity and sex.				

## Discussion

We found positive associations between density and proximity of natural gas wells within a 10-mile radius of maternal residence and birth prevalence of CHDs and possibly NTDs. Prevalence of CHDs increased monotonically from the lowest to highest exposure tertile, although even in the highest tertile the magnitude of the association was modest. Prevalence of NTDs was elevated only in the highest tertile of exposure. We also observed small negative associations between density and proximity of natural gas wells within a 10-mile radius of maternal residence and preterm birth and term low birth weight, and a small positive association with mean birth weight. We found no indication of an association between density and proximity of natural gas wells within a 10-mile radius of maternal residence and oral cleft prevalence.

Nongenetic risk factors for CHDs and NTDs possibly attributable to NGD include maternal exposure to benzene (Lupo et al. 2010b; Wennborg et al. 2005), PAHs (Ren et al. 2011), solvents (Brender et al. 2002; Chevrier et al. 1996; Desrosiers et al. 2012; McMartin et al. 1998), and air pollutants (NO_2,_ SO_2_, PM) (Vrijheid et al. 2011). NGD emits multiple air pollutants, including benzene and toluene, during the “well completion” phase (when gas and water flow back to the surface after hydraulic fracturing) as well as from related infrastructure (CDPHE 2009a, 2009b; Garfield County Public Health Department 2009; Gilman et al. 2013; McKenzie et al. 2012; Pétron et al. 2012). Ambient benzene levels in areas with active NGD in Northeast Colorado ranged from 0.03 to 6 parts per billion by volume (ppbv) (CDPHE 2012; Gilman et al. 2013; Pétron et al. 2012). Furthermore, 24-hr average ambient air benzene levels near active well development sites in western Colorado ranged from 0.03 to 22 ppbv (McKenzie et al. 2012).

Two previous case–control studies have reported associations between maternal exposure to benzene and birth prevalence of NTDs and/or CHDs (Lupo et al. 2010b; Wennborg et al. 2005). The study by Lupo et al. (2010b) of 4,531 births in Texas found that mothers living in census tracts with the highest ambient benzene levels (0.9–2.33 ppbv) were 2.3 times more likely to have offspring with spina bifida than mothers living in census tracts with the lowest ambient benzene levels (95% CI: 1.22, 4.33). An occupational study of Swedish laboratory employees found a significant association between exposure to occupational levels of benzene in the critical window between conception, organogenesis, and neural crest formation and neural crest malformations (Wennborg et al. 2005). Children born to 298 mothers exposed to benzene had 5.3 times greater prevalence of neural crest malformations than children born to mothers not exposed to benzene (95% CI: 1.4, 21.1). Other studies of maternal exposures to organic solvents, some of which contain benzene, have reported associations between maternal occupational exposure to organic solvents and major birth defects (Brender et al. 2002; Desrosiers et al. 2012; McMartin et al. 1998). Although exposure to benzene is a plausible explanation for the observed associations, further research is needed to examine whether these associations are replicated and whether benzene specifically explains these associations.

Air pollutants emitted from diesel engines used extensively in NGD also may be associated with CHDs and/or NTDs. Trucks with diesel engines are used to transport supplies, water, and waste to and from gas wells, with 40 to 280 truck trips per day per well pad during development (Witter et al. 2013). Generators equipped with diesel engines are used in both drilling wells and hydraulic fracturing. Air pollutants in diesel exhaust include NO_2_, SO_2_, PM, and PAHs. A meta-analysis of four studies suggested associations of maternal NO_2_ and SO_2_ exposures with coarctation of the aorta and tetralogy of Fallot, and of maternal PM_10_ exposure with arterial septal defects (Vrijheid et al. 2011). Two case–control studies in China reported positive associations between PAH concentrations in maternal blood and the placenta and NTDs (Li et al. 2011; Naufal et al. 2010). Several CHDs were associated with traffic related carbon monoxide and ozone pollution in a case control study of births from 1987 to 1993 in Southern California (Ritz et al. 2002).

The small negative associations with term low birth weight and preterm birth in our study population were unexpected given that other studies have reported postive associations between these outcomes and urban air pollution (Ballester et al. 2010; Brauer et al. 2008; Dadvand et al. 2013; Ghosh et al. 2012; Llop et al. 2010) and proximity to natural gas wells (Hill 2013). It is possible that rural air quality near natural gas wells in Colorado is not as compromised as urban air quality in these studies, and exposure represented as IDW well count may not adequately represent air quality. In addition, the power of our large cohort increases the likelihood of false positive results for small associations close to the null. Although associations were consistent across measures of birth weight (i.e., reduced risk of term low birth weight and increase in mean birth weight), they attenuated toward the null in sensitivity analysis for 2- and 5-mile radii (see Supplemental Material, Tables S6–S7). If causal, stronger associations would be expected with more stringent exposure definions. Our incomplete ability to adjust for socioeconomic status, health, nutrition, prenatal care, and pregnancy complications likely accounts for these unexpected findings.

This study has several limitations inherent in the nature of the available data. Not all birth defects were confirmed by medical record review. Also, birth defects are most likely undercounted, because stillbirths, terminated pregnancies, and later-life diagnoses (after 3 years of age) are not included. Birth weight and gestational age were obtained from birth certificates, which are generally accurate for birth weight and useful but less accurate for gestational age (DiGiuseppe et al. 2002). Data on covariates were obtained from birth certificates and were limited to basic demographic, education, and behavioral information available in the vital records. Distribution of covariates among exposure tertiles and the unexposed group was similar; nevertheless, our incomplete ability to adjust for socioeconomic status, health, nutrition, prenatal care, and pregnancy complications may have resulted in residual confounding. In addition, low event outcomes (e.g., NTDs) were adjusted only for elevation. The data set did not contain information on maternal folate consumption and genetic anomalies, both independent predictors of our outcomes, which may have confounded these results. We did observe a large decrease in the prevalence of NTDs after the introduction of folic acid in 1998, and small increases in the prevalence of CHDs and oral clefts, although none of the estimates are statistically significant (see Supplemental Material, Table S1). Further study is needed to determine whether unaccounted folate confounding is attenuating our results toward the null. There is no evidence indicating genetic anomalies would differ by IDW well count around maternal residence.

Because of the rarity of specific birth defects in the study population, birth defects were aggregated into three general groups. This limited our study in that associations with specific birth defects may have been obscured. An exploratory analysis of CHDs by clinical diagnostic groups indicates increased prevalence of specific diagnostic groups (i.e., PAV, VSD, and TVD) compared with aggregated CHDs (Table 4).

Another limitation of this study is the lack of temporal and spatial specificity of the exposure assessment. Because we did not have maternal residential history, we assumed that maternal address at time of delivery was the same as maternal address during the first trimester of pregnancy—the critical time period for formation of birth defects. Studies in Georgia and Texas estimate that 22–30% of mothers move residence during their pregnancy, and most mothers move within their locality (Lupo et al. 2010a; Miller et al. 2010), potentially introducing some exposure misclassification for the early pregnancy period of interest. However, these studies found little difference in mobility between cases and controls (Lupo et al. 2010a; Miller et al. 2010), and maternal mobility did not significantly influence the assessment of benzene exposure (Lupo et al. 2010a). We were able to determine only whether a well existed within the calendar year of birth (e.g., 2003) and did not have sufficient data to determine if a well existed within the first trimester of the pregnancy. Therefore, some nondifferential exposure misclassification is likely and the overall effect of this is unknown.

Similarly, we had consistent information only on existence of a well in the birth year. Lack of information on natural gas well activity levels, such as whether or not wells were producing or undergoing development, may have resulted in exposure misclassification. Actual exposure to natural gas–related pollutants likely varies by intensity of development activities. Lack of temporal and spatial specificity of the exposure assessment would most likely have tended to weaken associations (Ritz et al. 2007; Ritz and Wilhelm 2008). To address spatial and temporal variability, additional air pollution measurements and modeling will be needed to improve exposure estimates at specific locations. Last, information on the mother’s activities away from her residence, such as work and recreation, as well as proximity of these activities to NGD was not available and may have led to further exposure misclassification and residual confounding.

## Conclusion

This study suggests a positive association between greater density and proximity of natural gas wells within a 10-mile radius of maternal residence and greater prevalence of CHDs and possibly NTDs, but not oral clefts, preterm birth, or reduced fetal growth. Further studies incorporating information on specific activities and production levels near homes over the course of pregnancy would improve exposure assessments and provide more refined effect estimates. Recent data indicate that exposure to NGD activities is increasingly common. The COGCC estimates that 26% of the > 47,000 oil and gas wells in Colorado are located within 150–1,000 feet of a home or other type of building intended for human occupancy (COGCC 2012). Taken together, our results and current trends in NGD underscore the importance of conducting more comprehensive and rigorous research on the potential health effects of NGD.

## Supplemental Material

(143 KB) PDF
