# Supplementary material for: Birth Outcomes and Maternal Residential Proximity to Natural Gas Development in Rural Colorado
Source: Environ Health Perspect. 2014 Jan 28;122(4):412–7. doi: 10.1289/ehp.1306722 (PMC3984231; doi:10.1289/ehp.1306722)
Supplement: (143 KB) PDF [file ehp.1306722.s001.pdf]

Environmental Health Perspectives doi:10.1289/ehp.1306722

Birth Outcomes and Maternal Residential Proximity to Natural Gas Development in Rural Colorado

Lisa M. McKenzie, Ruixin Guo, Roxana Z. Witter, David A. Savitz, Lee S. Newman, and John L. Adgate

Table of Contents

Table S1. Association of Congenital Heart Defects, Neural Tube Defects, and Oral Clefts Before and After 1998 to Estimate Effect of Introduction of Folic Acid Fortification in 1998 **2**

Table S2. Association Between Inverse Distance Weighted Well Count within Two- and Five-Mile Radii of Maternal Residence and Ten-Mile Radius Births Between 2000-2009 and Congenital Heart Defects **3**

Table S3. Association Between Inverse Distance Weighted Well Count within Two- and Five-Mile Radii of Maternal Residence and Ten-Mile Radius Births Between 2000-2009 and Neural Tube Defects. **4**

Table S4. Association Between Inverse Distance Weighted Well Count within Two- and Five-Mile Radii of Maternal Residence and Ten-Mile Radius Births Between 2000-2009 and Oral Clefts. **5**

Table S5. Association Between Inverse Distance Weighted Well Count within Two- and Five-Mile Radii of Maternal Residence and Ten Mile Radius Births Between 2000-2009 and Preterm Birth. **6**

Table S6. Association Between Inverse Distance Weighted Well Count within Two- and Five-Mile Radii of Maternal Residence and Ten-Mile Radius Births Between 2000-2009 and Full Term Low Birth Weight. **7**

Table S7. Association Between Inverse Distance Weighted Well Count within Two- and Five-Mile Radii of Maternal Residence and Ten-Mile Radius Births Between 2000-2009 and Full Term Birth Weight. **8**

Table S1. Association of Congenital Heart Defects, Neural Tube Defects, and Oral Clefts Before and After 1998 to Estimate Effect of Introduction of Folic Acid Fortification in 1998[a].

Birth Outcome	Cases (n)	Crude OR (95% Cl)	Adjusted OR (95% Cl)
Congenital Heart Defects[b]	1823	1.1 (0.95, 1.3)	1.1 (0.97, 1.3)[b]
Neural Tube Defects[c]	59	0.67 (0.34, 1.3)	0.67 (0.34, 1.3)[c]
Oral Clefts[b]	251	1.1 (0.76, 1.7)	1.1 (0.76, 1.7)[b]

[a]Odds ratios represent the prevalence of each outcome after 1998 compared to births before 1998. Births in 1998 not included in analysis. [b]Adjusted for maternal age, ethnicity, smoking, alcohol use, education, and elevation of residence, as well as infant parity and gender. [c]Adjusted for elevation of maternal residence.

Table S2. Association Between Inverse Distance Weighted Well Count within Two- and Five-Mile Radii of Maternal Residence and Ten-Mile Radius Births Between 2000-2009 and Congenital Heart Defects.

Inverse Distance Weighted Well Count	Parameter	Two Mile Radius[a]	Five Mile Radius[b]	Ten Mile Radius 2000-2009[c]
**No Wells**	Live Births	97899	81649	48749
	Cases (n)	1351	1101	676
	Crude OR	1	1	1
**Low**	Live Births	8891	14253	14773
	Cases (n)	147	233	225
	Crude OR	1.2	1.2	1.1
	Adjusted OR (95% Cl)[d]	1.2 (0.97, 1.4)	1.2 (1.0, 1.4)	1.1 (0.93, 1.3)
**Medium**	Live Births	8892	14255	14772
	Cases (n)	161	236	237
	Crude OR	1.3	1.2	1.2
	Adjusted OR (95% Cl)[d]	1.3 (1.1, 1.6)	1.2 (1.1, 1.4)	1.2 (0.99, 1.4)
**High**	Live Births	9160	14685	15220
	Cases (n)	164	253	286
	Crude OR	1.3	1.3	1.4
	Adjusted OR (95% Cl)[d]	1.3 (1.1, 1.5)	1.3 (1.1, 1.5)	1.4 (1.2, 1.6)

[a]Low = first tertile, 1 to 156 wells per mile, medium = second tertile, 157 to 388 wells per mile, high = third tertile, 389 to 1400 wells per mile. [b]Low = first tertile, 1 to 20.1 wells per mile, medium = second tertile, 20.2 to 229 wells per mile, high = third tertile, 230 to 1400 wells per mile. [c]Low = first tertile, 1 to 3.87 wells per mile, medium = second tertile, 3.88 to 166 wells per mile, high = third tertile167 to 1400 wells per mile. [d]Adjusted for maternal age, ethnicity, smoking, alcohol use, education, and elevation of residence, as well as infant parity and gender

Table S3. Association Between Inverse Distance Weighted Well Count within Two- and Five-Mile Radii of Maternal Residence and Ten-Mile Radius Births Between 2000-2009 and Neural Tube Defects.

Inverse Distance Weighted Well Count	Parameter	Two-Mile Radius[a]	Five-Mile Radius[b]	Ten-Mile Radius 2000-2009[c]
**No Wells**	Live Births (N)	97899	81649	48749
	Cases (n)	39	31	21
	Crude OR	1	1	1
**Low**	Live Births (N)	8891	14253	14773
	Cases (n)	< 5[e]	5	5
	Crude OR	1.1	0.92	0.79
	Adjusted OR (95% Cl)[d]	1.0 (0.37, 3.0)	0.83 (0.31, 2.2)	0.65 (0.23, 1.8)
**Medium**	Live Births (N)	8892	14255	14772
	Cases (n)	8	12	6
	Crude OR	2.3	2.2	0.94
	Adjusted OR (95% Cl)[d]	2.1 (0.95, 4.5)	2.0 (1.0, 4.0)	0.82 (0.32, 2.1)
**High**	Live Births (N)	9160	14685	15220
	Cases (n)	8	11	11
	Crude OR	2.2	2.0	1.7
	Adjusted OR (95% Cl)[d]	1.9 (0.86, 4.3)	1.7 (0.81, 3.6)	1.4 (0.60, 3.1)

[a]Low = first tertile, 1 to 156 wells per mile, medium = second tertile, 157 to 388 wells per mile, high = third tertile, 389 to 1400 wells per mile. [b]Low = first tertile, 1 to 20.1 wells per mile, medium = second tertile, 20.2 to 229 wells per mile, high = third tertile, 230 to 1400 wells per mile. [c]Low = first tertile, 1 to 3.87 wells per mile, medium = second tertile, 3.88 to 166 wells per mile, high = third tertile, 167 to 1400 wells per mile. [d]Adjusted for elevation of maternal residence. [e]Counts of < 5 are not reported to protect identity of subjects.

Table S4. Association Between Inverse Distance Weighted Well Count within Two- and Five-Mile Radii of Maternal Residence and Ten-Mile Radius Births Between 2000-2009 and Oral Clefts.

Inverse Distance Weighted Well Count	Parameter	Two-Mile Radius[a]	Five-Mile Radius[b]	Ten-Mile Radius 2000-2009[c]
**No Wells**	Live Births (N)	97899	81649	48749
	Cases (n)	195	165	98
	Crude OR	1	1	1
**Low**	Live Births (N)	8891	14253	14773
	Cases (n)	19	27	25
	Crude OR	1.1	0.94	0.84
	Adjusted OR (95% Cl)[d]	0.99 (0.62, 1.6)	0.83 (0.54, 1.3)	0.73 (0.46, 1.2)
**Medium**	Live Births (N)	8892	14255	14772
	Cases (n)	22	34	26
	Crude OR	1.2	1.2	0.88
	Adjusted OR (95% Cl)[d]	1.2 (0.73, 1.8)	1.0 (0.70, 1.5)	0.77 (0.49, 1.2)
**High**	Live Births (N)	9160	14685	15220
	Cases (n)	15	25	31
	Crude OR	0.82	0.84	1.0
	Adjusted OR (95% Cl)[d]	0.76 (0.44, 1.3)	0.77 (0.49, 1.2)	0.87 (0.55, 1.4)

[a]Low = first tertile, 1 to 156 wells per mile, medium = second tertile, 157 to 388 wells per mile, high = third tertile, 389 to 1400 wells per mile. [b]Low = first tertile, 1 to 20.1 wells per mile, medium = second tertile, 20.2 to 229 wells per mile, high = third tertile, 230 to 1400 wells per mile. [c]Low = first tertile, 1 to 3.87 wells per mile, medium = second tertile, 3.88 to 166 wells per mile, high = third tertile167 to 1400 wells per mile. [d]Adjusted for maternal age, ethnicity, smoking, alcohol use, education, and elevation of residence, as well as infant parity and gender.

Table S5. Association Between Inverse Distance Weighted Well Count within Two- and Five-Mile Radii of Maternal Residence and Ten-Mile Radius Births Between 2000-2009 and Preterm Birth.

Inverse Distance Weighted Well Count	Parameter	Two-Mile Radius[a]	Five-Mile Radius[b]	Ten-Mile Radius 2000-2009[c]
**No Wells**	Live Births (N)	96230	80274	47914
	Cases (n)	6994	5887	3642
	Crude OR	1	1	1
**Low**	Live Births (N)	8722	13983	14513
	Cases (n)	604	1086	1046
	Crude OR	0.95	1.1	0.94
	Adjusted OR (95% Cl)[d]	0.97 (0.89, 1.1)	1.1 (0.99, 1.1)	0.91 (0.84, 0.99)
**Medium**	Live Births (N)	8701	13976	14502
	Cases (n)	517	820	956
	Crude OR	0.8	0.79	0.86
	Adjusted OR (95% Cl)[d]	0.86 (0.79, 0.95)	0.83 (0.76, 0.89)	0.87 (0.80, 0.94)
**High**	Live Births (N)	8975	14395	14893
	Cases (n)	655	977	993
	Crude OR	1.0	0.92	0.87
	Adjusted OR (95% Cl)[d]	1.0 (0.94, 1.1)	0.96 (0.89, 1.0)	0.88 (0.81, 0.96)

[a]Low = first tertile, 1 to 156 wells per mile, medium = second tertile, 157 to 388 wells per mile, high = third tertile, 389 to 1400 wells per mile. [b]Low = first tertile, 1 to 20.1 wells per mile, medium = second tertile, 20.2 to 229 wells per mile, high = third tertile, 230 to 1400 wells per mile. [c]Low = first tertile, 1 to 3.87 wells per mile, medium = second tertile, 3.88 to 166 wells per mile, high = third tertile, 167 to 1400 wells per mile. [d]Adjusted for maternal age, ethnicity, smoking, alcohol use, education, and elevation of residence, as well as infant parity and gender.

Table S6. Association Between Inverse Distance Weighted Well Count within Two- and Five-Mile Radii of Maternal Residence and Ten-Mile Radius Births Between 2000-2009 and Full Term Low Birth Weight.

Inverse Distance Weighted Well Count	Parameter	Two-Mile Radius[a]	Five-Mile Radius[b]	Ten-Mile Radius 2000-2009[c]
**No Wells**	Live Births (N)	89231	74382	44268
	Cases (n)	3129	2785	1683
	Crude OR	1	1	1
**Low**	Live Births (N)	8118	12897	13466
	Cases (n)	189	391	395
	Crude OR	0.66	0.79	0.77
	Adjusted OR (95% Cl)[d]	0.80 (0.68, 0.93)	0.96 (0.86, 1.1)	0.97 (0.86, 1.1)
**Medium**	Live Births (N)	8181	13154	13546
	Cases (n)	189	327	360
	Crude OR	0.65	0.66	0.69
	Adjusted OR (95% Cl)[d]	0.86 (0.74, 1.0)	0.88 (0.77, 0.99)	0.86 (0.76, 0.97)
**High**	Live Births (N)	8317	13414	13896
	Cases (n)	209	342	330
	Crude OR	0.71	0.65	0.62
	Adjusted OR (95% Cl)[d]	0.99 (0.85, 1.2)	0.93 (0.81, 1.1)	0.89 (0.78, 1.0)

[a]Low = first tertile, 1 to 156 wells per mile, medium = second tertile, 157 to 388 wells per mile, high = third tertile, 389 to 1400 wells per mile. [b]low = first tertile, 1 to 20.1 wells per mile, medium = second tertile, 20.2 to 229 wells per mile, high = third tertile, 230 to 1400 wells per mile. [c]Low = first tertile, 1 to 3.87 wells per mile, medium = second tertile, 3.88 to 166 wells per mile, high = third tertile167 to 1400 wells per mile. [d]Adjusted for maternal age, ethnicity, smoking, alcohol use, education, and elevation of residence, as well as infant parity, gender, and gestational age.

Table S7. Association Between Inverse Distance Weighted Well Count within Two- and Five-Mile Radii of Maternal Residence and Ten-Mile Radius Births Between 2000-2009 and Full Term Birth Weight.

Inverse Distance Weighted Well Count	Parameter	Two-Mile Radius[a]	Five-Mile Radius[b]	Ten-Mile Radius 2000-2009[c]
**No Wells**	Live Births (N)	89231	74382	44268
	Mean difference in birth weight (grams)[d]	0	0	0
**Low**	Live Births (N)	8118	12897	13466
	Mean difference in birth weight (grams)[d]	29 (20, 39)	21 (13, 29)	4.2 (-4.2, 13)
**Medium**	Live Births (N)	8181	13154	13546
	Mean difference in birth weight (grams)[d]	20 (11, 30)	28 (20, 35)	19 (11, 27)
**High**	Live Births (N)	8317	13414	13896
	Mean difference in birth weight (grams)[d]	15 (5, 24)	18 (10, 26)	25 (17, 34)

[a]Low = first tertile, 1 to 156 wells per mile, medium = second tertile, 157 to 388 wells per mile, high = third tertile, 389 to 1400 wells per mile. [b]Low = first tertile, 1 to 20.1 wells per mile, medium = second tertile, 20.2 to 229 wells per mile, high = third tertile, 230 to 1400 wells per mile. [c]Low = first tertile, 1 to 3.87 wells per mile, medium = second tertile, 3.88 to 166 wells per mile, high = third tertile, 167 to 1400 wells per mile. [d]Adjusted for maternal age, ethnicity, smoking, alcohol use, education, and elevation of residence, as well as infant parity, gender, and gestational age.
